# The FDA-approved natural product dihydroergocristine reduces the production of the Alzheimer’s disease amyloid-β peptides

**DOI:** 10.1038/srep16541

**Published:** 2015-11-16

**Authors:** Xiling Lei, Jing Yu, Qi Niu, Jianhua Liu, Patrick C. Fraering, Fang Wu

**Affiliations:** 1 Key Laboratory of Systems Biomedicine (Ministry of Education), Shanghai Center for Systems Biomedicine, Shanghai Jiao Tong University, Shanghai, China; 2 State Key Laboratory of Microbial Metabolism & School of Life Sciences and Biotechnology, Shanghai Jiao Tong University, Shanghai, China; 3 Brain Mind Institute, School of Life Sciences, Ecole Polytechnique Federale de Lausanne (EPFL), Lausanne, Switzerland

## Abstract

Known γ-secretase inhibitors or modulators display an undesirable pharmacokinetic profile and toxicity and have therefore not been successful in clinical trials for Alzheimer’s disease (AD). So far, no compounds from natural products have been identified as direct inhibitors of γ-secretase. To search for bioactive molecules that can reduce the amount of amyloid-beta peptides (Aβ) and that have better pharmacokinetics and an improved safety profile, we completed a screen of ~400 natural products by using cell-based and cell-free γ-secretase activity assays. We identified dihydroergocristine (DHEC), a component of an FDA- (Food and Drug Administration)-approved drug, to be a direct inhibitor of γ-secretase. Micromolar concentrations of DHEC substantially reduced Aβ levels in different cell types, including a cell line derived from an AD patient. Structure-activity relationship studies implied that the key moiety for inhibiting γ-secretase is the cyclized tripeptide moiety of DHEC. A Surface Plasmon Resonance assay showed that DHEC binds directly to γ-secretase and Nicastrin, with equilibrium dissociation constants (K_d_) of 25.7 nM and 9.8 μM, respectively. This study offers DHEC not only as a new chemical moiety for selectively modulating the activity of γ-secretase but also a candidate for drug repositioning in Alzheimer’s disease.

Alzheimer’s disease (AD) is the most common neurodegenerative disease among elderly people worldwide^1^,^2^. Unfortunately, no disease-modifying drugs are currently available, and it is unlikely that any will enter the market in the near future^1^,^3^.

The exact sequence of events in the pathogenesis of AD remains unknown, although several mechanisms have been proposed^4^. The most popular amyloid hypothesis suggests that the occurrence of AD is linked to abnormal amyloid-β (Aβ) production, oligomerization or clearing, which are complex processes that offer several opportunities for therapeutic intervention^5^. Aβ generation and the profiles of Aβ peptides (from 38 to 43 amino acids long) in different species are controlled by the γ-secretase-mediated proteolysis of the amyloid-β precursor protein (APP)^6^. Thus, inhibition or modulation of γ-secretase activity is considered to be an important therapeutic approach for the treatment of AD^3^.

Diverse classes of γ-secretase inhibitors (GSI) or modulators (GSM) have been discovered for lowering Aβ peptides or modulating their composition^7^,^8^. The success of some γ-secretase inhibitors or modulators has been prevented by low efficacy, poor blood–brain barrier penetration or severe side effects^8^,^9^,^10^,^11^. To improve the therapeutic benefits of GSI or GSM, it is crucial to find new chemical moieties that have safer and better pharmacokinetics profiles^11^,^12^. Seeking new chemical skeletons from natural products that could reduce the Aβ level is one method that researchers are currently pursuing^12^,^13^. However, no pure compound that can directly inhibit the activity of γ-secretase has been identified from natural products.

In this study, we screened 417 natural products in our γ-secretase assays and identified that the natural product dihydroergocristine (DHEC) suppresses the production of Aβ peptides in cell-based and cell-free *in vitro* purified γ-secretase assays. DHEC is a component of ergoloid mesylates, a US Food and Drug Administration (FDA)-approved prescription drug for the treatment of hypertension and dementia, and ergoloid mesylates shows no severe side-effects according to the 34^th^ edition of The Orange Book and the description on the drug label^14^,^15^,^16^.

## Results

### Dihydroergocristine inhibits cellular Aβ production and the activity of γ-secretase, without affecting the processing of the Notch receptor

To identify natural product-based bioactive inhibitors of γ-secretase, we screened 417 natural products by using a cell-based luciferase reporter assay for γ-secretase inhibition (T_100_, see Methods), which we recently developed in TREx HeLa cells according to the methodology described in ref. 17. In this cellular assay, the well-known γ-secretase inhibitor DAPT showed dose-dependent inhibition of APP-C99 processing, with an IC_50_ value of ~200 nM (Supplementary Fig. S1a). Thus, this assay is sufficiently sensitive to detect the inhibitory effects of 100 nM DAPT on cellular γ-secretase activity. Additionally, this new cell-based assay tolerated up to 2% DMSO, which is a great advantage when screening inhibitors in a high-throughput format.

**Figure 1:**
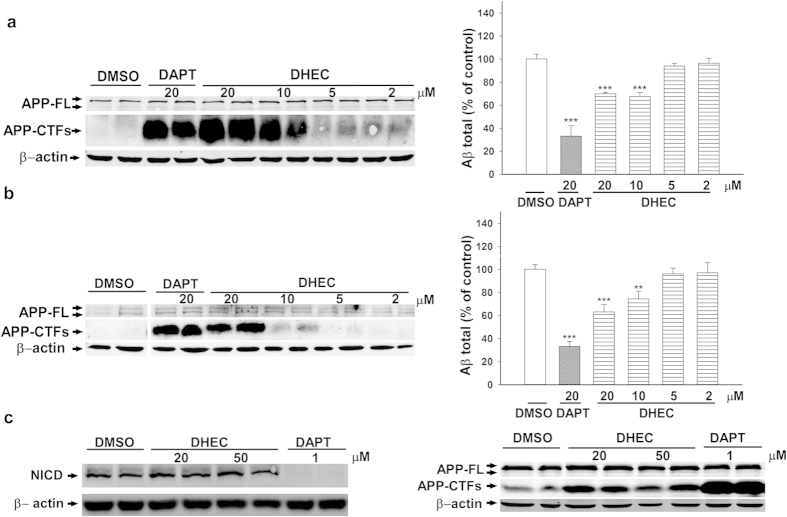
Dihydroergocristine inhibits the intracellular production of Aβ and the activity of γ-secretase, without affecting Notch processing. (**a**) Effects of DHEC on endogenous APP-CTF accumulation and Aβ generation in HEK293 cells. HEK293 cells were incubated with DMSO (control), the indicated concentrations of DHEC or 20 μM DAPT in 24-well plates for 24 h, before Western blot analysis of APP-FL and APP-CTF (left panel). The levels of β-actin were used as equal loading controls. The corresponding media from the DMSO-, DHEC- or DAPT- treated groups (n = 3) were collected, and the Aβ total level was measured by ELISA (right panel). The Aβ data are expressed as a percentage of the control value and presented as the means ± sd. Asterisks indicate significant differences (***P < 0.001; one-way ANOVA with Bonferroni’s multiple comparisons tests) in Aβ total production of the treated samples compared with the controls (DMSO). (**b**) Effects of DHEC on endogenous γ-secretase activity in fibroblast cells from an AD patient. Fibroblast cells from an AD patient carrying the PS1 missense mutation A246E were treated with various compounds, and the levels of APP-FL, APP-CTF and β-actin, as well as Aβ, were measured as described above. The Aβ data are expressed as a percentage of the control value and presented as the means ± sd. (n = 3). Asterisks indicate significant differences (**P < 0.01; ***P < 0.001; one-way ANOVA with Bonferroni’s multiple comparisons tests) in Aβ total production of the samples compared with the controls (DMSO). (**c**) Effects of DHEC on the cleavage of human Notch1 and APP in HEK293 cells overexpressing the human Notch1 extracellular truncation (NEXT; left panel) and APP (right panel), respectively. After 24  h transient transfection of HEK293 cells with Notch1 NEXT or hAPP plasmids, cells were incubated with DHEC or DAPT at the indicated concentrations for one additional day before Western Blot analysis of NICD (Ab1744) and APP-CTF (C-T15). The levels of β-actin served as equal loading controls. The densitometric quantifications for the Western Blots are shown in Supplementary Fig. S2. For full blots, please see Supplementary Fig. S9.

**Figure 2:**
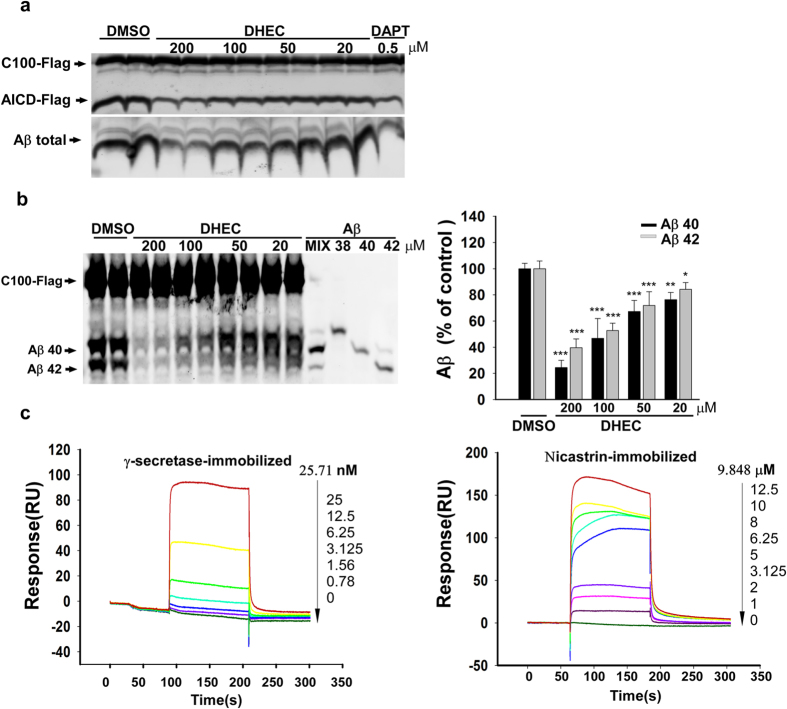
Effects of dihydroergocristine on the processing of human APP C100-Flag. (**a**) Dose-dependent effects of DHEC on the cleavage of C100-Flag by purified γ-secretase. Purified γ-secretase solubilized in 0.2% CHAPSO-HEPES was incubated at 37 °C for 4 h with 1 μM C100-Flag substrate, 0.1% PC, and the indicated concentrations of dihydroergocristine (DHEC) or DMSO (control, 100%). Reactions were stopped by adding 0.5% SDS, and the resultant products were separated in 16% Tricine-SDS-PAGE gels, transferred to a membrane and detected with anti-AICD-Flag antibody (C-T15) and anti-Aβ antibody (6E10; lower panel). The density of the AICD-Flag and Aβ total bands was quantified by Odyssey software (Supplementary data Fig. S5). (**b**) Dose-dependent effects of DHEC on Aβ40 and Aβ42 production in the cell-free assay performed with purified C100-Flag and γ-secretase. Reactions in the *in vitro* assay upon treatment with DHEC, using purified γ-secretase and C100-Flag, were tested as described above. The residue was then separated by Bicine/urea SDS-PAGE, together with an Aβ standard of synthetic human Aβ38, Aβ40 and Aβ42, followed by Western blot detection with anti-Aβ antibody (6E10, left panel), or quantification by ELISA (right panel). Black bars, Aβ [1–40]; gray bars, Aβ[1–42]. Data were presented as the means ± sd. (n = 3) Asterisks indicate significant differences (**P < 0.01; ***P < 0.001; two-way ANOVA with Bonferroni’s multiple comparisons tests) in Aβ40 or Aβ42 production of DHEC-treated samples compared with the control (DMSO). (**c**) Surface plasmon resonance assay analysis of the binding of DHEC to γ-secretase or NCT. Solutions of various concentrations of DHEC were injected into the chamber with a γ-secretase (left panel) or NCT (right panel)-coated sensor chip. The change in response units over time is shown.

After primary screening of the compounds in T_100_ cells, a total of 8 natural products were found to inhibit the cellular activity of γ-secretase, in a dose-dependent manner and with an IC_50_ < 30 μM. Of these, NSC409663 (DHEC), which was identified from the natural product library of the National Cancer Institute (NCI, Bethesda, USA), was the only compound that affected the activity of γ-secretase in both cell-based and cell-free assays (Figs 1 and 2). DHEC, which has been used for the treatment of glaucoma^18^, is also a component of the drug ergoloid mesylates. Ergoloid mesylates contains a mixture of four ergot alkaloids (DHEC, dihydroergocornine, α-dihydroergocryptine and β–dihydroergocryptine; refs 14,15). In our study, DHEC had an IC_50_ value of ~25 μM for inhibiting the activity of γ-secretase in T_100_ cells without affecting cell viability (Supplementary Fig. S1b). In HEK293 cells, DHEC also caused a significant dose-dependent accumulation of the carboxy-terminal fragments of APP (APP-CTFs, Fig. 1a; left panel; Supplementary Fig. S2a), and 10 μM DHEC resulted in a ~30% reduction in Aβ production (Fig. 1a; right panel), which did not influence the levels of full length APP (APP-FL) or cell viability at all tested doses (Fig. 1a, left panel; Supplementary Fig. S1c), as expected from a γ-secretase inhibitor^19^. Furthermore, 20 μM DHEC caused the accumulation of APP-CTFs and led to ~35% reduction in total Aβ (Fig. 1b; Supplementary Fig. S2b) in fibroblast cells from an Alzheimer’s disease patient carrying a missense mutation (A246E) in the presenilin 1 (PS1) gene. As predicted, DAPT caused a dose-dependent accumulation of APP-CTFs in HEK293 (Supplementary Fig. S3a) and fibroblast (Supplementary Fig. S3b) cells. Similarly, total Aβ levels were markedly reduced by treatment of both HEK293 and fibroblast cells with DAPT (Supplementary Fig. S3a,b and Fig. 1a,b, right panels).

To investigate whether the Aβ-lowering effect caused by DHEC can be attributed to changes in the expression levels of γ-secretase (the key enzyme responsible for Aβ production), whole extracts of HEK293 or fibroblast cells treated with either DHEC or control treatments (DMSO, negative control; DAPT, positive control) were analyzed by Western blotting for subunits of the protease complex. As indicated by the presence of the mature forms of the γ-secretase subunits Nicastrin (mNCT) and N-terminal fragment of PS1 (PS1-NTF)^20^, the assembly and maturation of the protease complex were not altered upon treatment with DHEC or DAPT (Supplementary Fig. S4). Together, these findings demonstrate that the reduced Aβ levels measured after DHEC treatment cannot be attributed to altered expression levels of γ-secretase subunits.

We next studied the effect of DHEC on the intracellular processing of the Notch1 receptor, a critical γ-secretase substrate implicated in different cell-fate decisions and the blocking of which results in clinical gastrointestinal side effects^7^. In HEK293 cells overexpressing an extracellularly truncated form of human Notch (NEXT), 20 or 50 μM DHEC did not inhibit the cleavage of this substrate and the production of the Notch intracellular domain (NICD, Fig. 1c left panel; Supplementary Fig. S2c left panel). In contrast, the same concentrations of DHEC substantially prevented the cleavage of APP-CTFs in HEK293 cells overexpressing hAPP-FL (Fig. 1c right panel; Supplementary Fig. S2c right panel). DAPT, a non-selective inhibitor of γ-secretase, showed no preference for inhibiting the processing of APP or Notch-based substrates (Supplementary Fig. S3c), and 1 μM DAPT completely blocked the production of intracellular human NICD (Fig. 1c left panel). Together, these data indicate that, in cells, DHEC preferentially inhibits the cleavage of an APP-based substrate rather than a Notch-based substrate.

### The γ-secretase-mediated processing of APP is inhibited by dihydroergocristine in assays with purified enzyme

To assess whether DHEC is a direct γ-secretase inhibitor, the compound was tested in a cell-free assay performed with purified γ-secretase and C100-Flag, a recombinant APP-CTF^21^. As shown in Fig. 2a, DHEC inhibited the γ-secretase-dependent processing of C100-Flag into AICD-Flag (a Flag-tagged APP intracellular domain) or into Aβ (Fig. 2a and Supplementary Fig. S5). Aβ40 and Aβ42 production were also inhibited, with an IC_50_ value of ~100 μM (Fig. 2b). As determined by Aβ ELISA or Western blotting of bicine/urea SDS-PAGE gels, DHEC treatment did not significantly change the ratio between Aβ40 and Aβ42, indicating that DHEC is a pan inhibitor of the generation of Aβ species of various lengths. Next, we used surface plasmon resonance (SPR; Biacore) to investigate whether DHEC interacts directly with the γ-secretase complex or with NCT, one of its subunits. SPR assays showed that DHEC binds directly to γ-secretase and to a lesser extent to NCT, with equilibrium dissociation constants (K_d_) of 25.7 nM and 9.8 μM, respectively (Fig. 2c). The K_d_ for the binding of DHEC to γ-secretase (25.7 nM) is much lower than the IC_50_ values of DHEC in the cellular and cell-free assays (20 and 100 μM, respectively). This result suggests that DHEC might bind to a site that overlaps with the APP substrate binding site, indicating that DHEC is a competitive inhibitor towards the substrate APP. In support of this idea, and consistent with our observation that DHEC binds to NCT with a K_d_ of ~10 μM (Fig. 2c), the APP binding site of γ-secretase has been proposed to be localized in the NCT subunit^22^. Taken together, these findings showed that DHEC might bind to NCT, with the possibility of having an additional binding site in one or more subunits of the γ-secretase complex.

### Structural requirements of dihydroergocristine for suppressing the activity of γ-secretase

**Figure 3:**
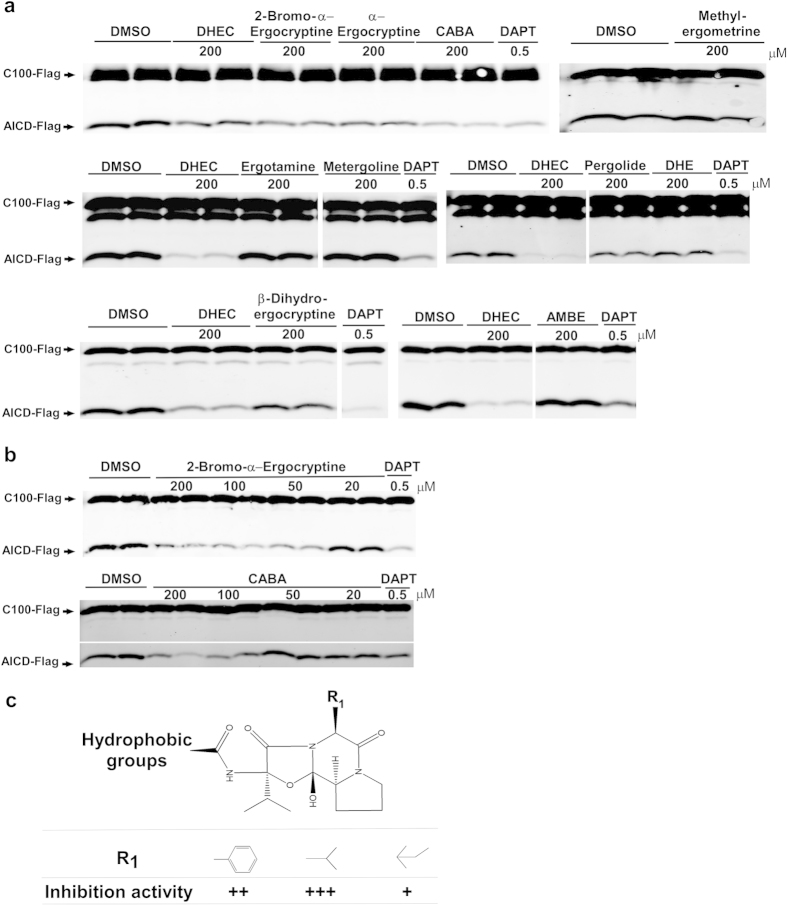
Structural requirements of dihydroergocristine for suppressing the activity of γ-secretase. (**a**) Effect of 200 μM dihydroergocristine (DHEC) analogs on C100-Flag cleavage by purified γ-secretase. The blots were processed under the same experiment conditions and each blot contained negative (DMSO) and positive (0.5 μM DAPT) controls as well as 200 μM DHEC. For full-length blots, please see Supplementary Fig. S10. (**b**) Dose-dependent effects of 2-bromo-α-ergocryptine and CABA on C100-Flag processing by purified γ-secretase. (**c**) Structure-activity relationship of DHEC analogs for inhibiting γ-secretase. The relative levels of AICD-Flag were estimated by densitometry and the quantification data are shown in Supplementary Fig. S6.

To identify the minimal core structure of DHEC that is responsible for suppressing the activity of γ-secretase, we next tested commercially available structural analogs of DHEC in our assay with purified γ-secretase (Table 1 and Fig. 3a). α-Ergocryptine is the closest analog of α-dihydroergocryptine, a component of ergoloid mesylates, while β-dihydroergocryptine is the other component of ergoloid mesylates^14^, both of which have similar chemical structures to DHEC (Table 1). Both 200 μM α–ergocryptine and 200 μM β-dihydroergocryptine inhibited the activity of γ-secretase (Fig. 3a; Supplementary Fig. S6a). DHEC, α-ergocryptine and β-dihydroergocryptine all contain a dimethyl group at the R_2_ position (corresponding to the side chain of valine in all three molecules) and a hydrophobic group at the R_1_ position (corresponding to the side chains phenylalanine, isoleucine and leucine, respectively; Table 1). In contrast, close analogs of DHEC, i.e., ergotamine and dihydroergotamine (DHE), both of which contain a methyl group at the R_2_ position instead of the dimethyl group in DHEC, did not inhibit γ-secretase activity (Table 1, Fig. 3a and Supplementary Fig. S6a).

Furthermore, three drugs (200 μM metergoline, pergolide and methylergometrine; Table 1) that contain only the lysergic acid moiety but not the cyclized tripeptide moiety did not inhibit γ-secretase (Table 1, Fig. 3a and Supplementary Fig. S6a). These results indicate that the cyclized tripeptide moiety is crucial for maintaining the inhibitory effects of this type of inhibitor, and for inhibition this moiety preferentially has a hydrophobic group at the R_1_ position and requires a dimethyl group at the R_2_ position. In addition to this, bromo substituted α-ergocryptine (200 μM) retained the ability to inhibit the activity of γ-secretase, indicating that additional modification at the lysergic acid moiety of these inhibitors is permitted. The IC_50_ of 2-bromo-α-ergocryptine in the *in vitro* γ-secretase activity assay was ~50 μM (Fig. 3b; Supplementary Fig. S6b). This was, in our hands, the most potent inhibitor of this type *in vitro*. To confirm that the cyclized tripeptide moiety is sufficient for inhibiting γ-secretase, we tested the compound CABA, which consists of the cyclized tripeptide part of DHEC with the side chain of valine at the R_1_ and R_2_ positions (Table 1). CABA showed dose-dependent inhibition of the activity of γ-secretase and an IC_50_ of ~100 μM (Table 1, Fig. 3a,b and Supplementary Fig. S6), which is comparable to that of DHEC (Fig. 2a), implying that only this moiety is needed for inhibiting the activity of γ-secretase. We also tested a non-cyclized tripeptide analog of CABA, namely AMBE, which clearly did not show any inhibitory activity (Table 1, Fig. 3a and Supplementary Fig. S6a).

Taken together, our results suggest that the cyclized tripeptide structure might be the minimally sufficient structural moiety for suppressing the activity of γ-secretase, with a Val at the R_2_ position and an unusual cyclol proline being indispensable and a Phe or Leu at the R_1_ position being preferred (Table 1 and Fig. 3c). Although 2-bromo-α-ergocryptine and CABA are comparable or better inhibitors of γ-secretase than is DHEC, these two compounds were not better inhibitors of APP cleavage in cells than was DHEC (Supplementary Fig. S7). In HEK293 cells overexpressing hAPP, 20 or 50 μM 2-bromo-α-ergocryptine was inactive, whereas CABA caused accumulation of APP-CTFs only when administered at a concentration of 50 μM (Supplementary Fig. S7).

## Discussion

Despite the growing number of AD patients, no disease-modifying therapies exist to safely treat this neurodegenerative disorder. The strategy of drug repositioning would accelerate drug research and development by rapidly providing available drugs for diseases^2^. In the present study, we have identified an FDA-approved drug, dihydroergocristine (DHEC), that can inhibit the production of Aβ *in vitro* and in cells (Table 1). DHEC is a component of ergoloid mesylates, also known as Hydergine, an FDA-approved drug that is clinically used for the treatment of idiopathic decline and hypertension^14^,^15^,^16^,^18^,^23^.

Ergoloid mesylates was introduced to clinical medicine in 1949 and has mainly been used for the treatment of dementia^24^. The effects of ergoloid mesylates were investigated in dozens of clinical trials between 1950 and 1990. Some clinical trials showed a positive effect, as evaluated by the outcome of global or comprehensive behavior ratings^24^,^25^ and SCAG (Sandoz Clinical Assessment-Geriatric Scale, ref. 15). Patients who suffer from diseases such as primary progressive dementia, Alzheimer’s dementia, senile onset dementia and multi-infarct dementia appear to respond to treatment with ergoloid mesylates according to descriptions of this drug^14^,^15^. Modest but statistically significant changes have been observed in mental alertness, confusion, recent memory, orientation, emotional lability, self-care, depression, anxiety/fears, cooperation, sociability, appetite, dizziness, fatigue, and bothersome (ness), as well as an overall improvement in clinical status. However, other clinical studies with ergoloid mesylates showed no benefit to patients^24^,^25^,^26^. Limitations in the design of clinical trials, such as the selection of patients and the diagnostic tools for dementia that were available at that time, probably explain the conflicting findings and therefore the lack of a clear conclusion about the efficacy of ergoloid mesylates in AD. The outcome of these clinical investigations indicated that the potentially effective doses of ergoloid mesylates may be higher than those currently approved, i.e., 3 mg daily in the United States^24^. Ergoloid mesylates, which has been prescribed for use even at a dose of 12 mg per day in some other countries^18^, is fairly well tolerated and safe for patients^24^,^26^. Given that DHEC reduced cellular Aβ levels when administered at micromolar concentrations as demonstrated in the present study, it seems worthwhile to retest the efficacy of ergoloid mesylates in pre-clinical or clinical studies at high doses and with updated clinical designs and tools for assessing AD. Such tools include the quantification of Aβ concentrations in the cerebrospinal fluid and the imaging of Aβ plaques with the latest generation of tracers^2^,^27^.

Ergoloid mesylates is a mixture of natural products and is composed of four compounds that are analogs of each other^28^. We have tested two components (DHEC and β-dihydroergocryptine) and two close analogs (α-ergocryptine and 2-bromo-α-ergocryptine) of α-dihydroergocryptine, another component of ergoloid mesylates. All of these compounds inhibited Aβ production in the γ-secretase assay performed with purified enzyme; the anti-pituitary and Parkinson’s disease drug 2-bromo-α-ergocryptine^18^ was the most effective, with an IC_50_ value of ~50 μM. After testing different close structural analogs of DHEC, we identified the cyclized tripeptide to be the minimally sufficient core moiety for inhibiting the activity of γ-secretase. These drugs are mainly modulators of the alpha adrenergic receptor and have a common lysergic acid moiety (Table 1; ref. 18). However, the lysergic acid moiety is also found in the inactive compounds investigated in the present study (ergotamine, dihydroergotamine, metergoline pergolide and methylergometrine), indicating that the structural core (lysergic acid moiety) of these receptor blockers is not sufficient for the γ-secretase inhibitory effects *in vitro* (Fig. 3). In contrast, the cyclized tripeptide CABA, which does not contain the lysergic acid moiety, is the smallest γ-secretase inhibitor amongst the tested compounds (Table 1).

**Figure 4:**
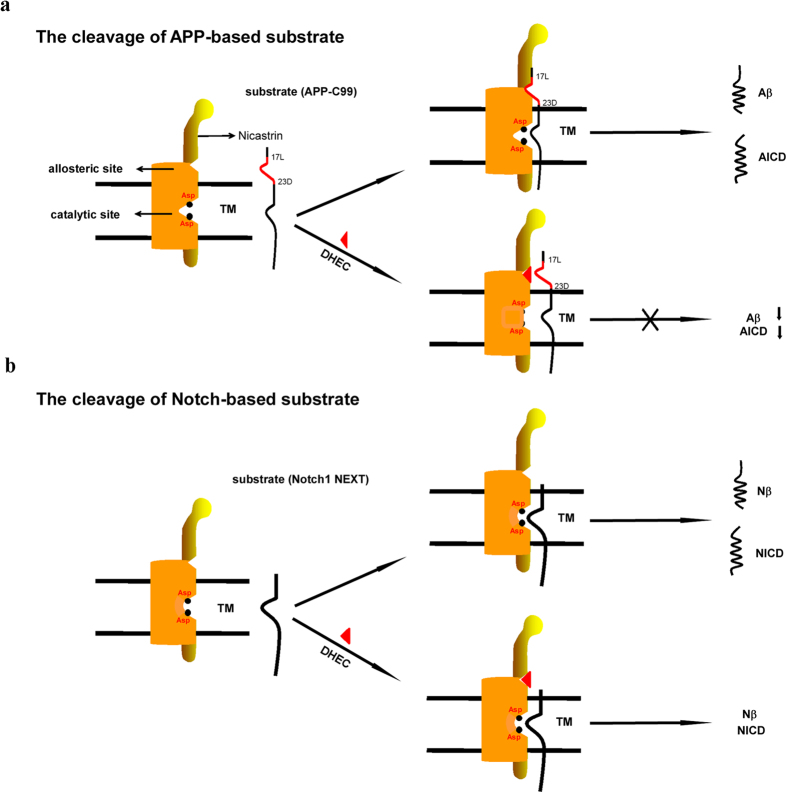
Hypothetical model for the selective DHEC-mediated inhibition of APP or Notch substrate cleavage by γ-secretase. (**a**) DHEC (red triangle) contains a Leu-Val-Phe motif (part of a known APP inhibitory domain (red line), which binds to an allosteric site made by both NCT (yellow) and PS1 (brown). Upon the binding to γ-secretase, DHEC will compete with the APP-C99 substrate for binding to this allosteric site, thus affecting the processing of APP-C99. (**b**) Because Notch1-NEXT is shorter than APP-C99 at the N-terminus of the extracellular domain and does not contain the known inhibitory motif, the binding of DHEC to γ-secretase does not affect the cleavage of this substrate.

The K_d_ for the binding of DHEC to γ-secretase (25.7 nM) is much lower than the IC_50_ values of DHEC in the cellular and cell-free assays (20 and 100 μM, respectively). This result suggests that DHEC might bind to a site that overlaps with the substrate binding site of APP and that the inhibitory effects of DHEC could be reduced by increasing the concentration of APP substrate (Fig. 4). To explain this observation, we measured *in vitro* the effect of DHEC on the cleavage of APP-C100 in the presence of a high concentration of C100-Flag substrate in the cell-free γ-secretase assay performed with purified enzyme. DHEC showed a greatly reduced inhibitory effect on γ-secretase in the presence of a high concentration of APP-C100 (4 μM; Supplementary Fig. S8), when compared to a lower concentration of APP-C100 (1 μM, Fig. 2a and Supplementary Fig. S5). This effect indicates that APP competes with γ-secretase for binding DHEC, thus reducing DHEC’s γ-secretase inhibitory activity. The binding site of APP has been hypothesized to be located in the NCT subunit^22^. Consistent with this hypothesis, our data show that DHEC binds to NCT with a K_d_ of 10 μM (Fig. 2c, right panel), implying that the binding site of DHEC could be partially located on NCT, while possibly having an additional site in one or more subunits of the γ-secretase complex. CABA, the minimal core structure, has the side chains of Leu and Val as well as a phenyl modification at the N-terminus; these functional groups could potentially mimic the side chains of Leu-Val-Phe at amino acids 17–19 of Aβ. The Leu-Val-Phe sequence has recently been proposed as the APP inhibitory domain, and shown to bind to an allosteric site in PS1, suggesting that DHEC may also bind to PS1^29^. Taken together, these data may suggest that DHEC binds to an allosteric site at the junction of the NCT and PS1 subunits of γ-secretase, which can be accessible to the Leu-Val-Phe motif of APP but not to the Notch substrate (Fig. 4). Thus, such an inhibitor could selectively block the cleavage of APP and reduce the production of Aβ and AICD without influencing the cleavage of Notch.

Because DHEC is tolerated by patients, is free of gastrointestinal toxicity and seems to have a beneficial therapeutic effect on dementia in clinical practice, we propose to investigate its effects on Aβ levels, cognition and behavior in preclinical or clinical studies.

In summary, we identified that the FDA-approved natural product DHEC effectively inhibited Aβ production in both cell-free and cell-based γ-secretase assays. The newly identified cyclized tripeptide structure of DHEC may serve as a better pharmacophore scaffold for developing new drugs for AD. Additionally, DHEC, an FDA-approved drug, might be considered as a candidate for drug repositioning to accelerate the development of treatments for AD.

## Methods

### Chemicals and reagents

Dihydroergocristine methanesulfonate salt, dihydroergotamine methanesulfonate salt, ergotamine tartrate, 2-bromo-α-ergocryptine methanesulfonate salt, metergoline, pergolide mesylate salt and DAPT (*N*-[*N*-(3,5-difluorophenylacetyl)-L-alanyl-]-(*S*)-phenylglycine-*t*-butyl ester) were purchased from Sigma-Aldrich (Steinheim, Germany). β-Dihydroergocryptine and α-ergocryptine were obtained from Johns Hopkins Clinical Compound Library (JHCCL, Baltimore, MD, USA; ref. 23), and methylergometrine (NSC186067) from the National Cancer Institute (NCI; Bethesda, MD). CABA([2R-(2α, 5α, 10aβ, 10bα)]-[Octahydro-10b-hydroxy-2-(1-methylethyl)-5-(2-methylpropyl)-3,6-dioxo-8H-oxazolo[3,2-a]pyrrolo[2,1-c]pyrazin-2-yl]-carbamic acid) was bought from Toronto Research Chemicals Inc. (North York, Canada) and tetracycline from Applichem (Darmstadt, Germany). AMBE ((S)-2-acetamido-3-methyl-N-[(S)-1-oxo-3-phenyl-1-(pyrrolidin-1-yl)propan-2-yl])butanamide) was synthesized by GL Biochem Ltd. (Shanghai, China). Protease inhibitors and X-tremeGENE HP DNA Transfection Reagent were obtained from Roche (Basel, Switzerland), Glo lysis buffer, Bright-Glo luciferase assay reagents and CytoTox-One^Tm^ kit were purchased from Promega (Madison, WI, USA). The BCA protein assay kit was purchased from Pierce Chemical (Rockford, IL, USA) and human Beta Amyloid [1-x], [1–40] or [1–42] colorimetric ELISA Kits from IBL (Gunma, Japan).

### Natural product library

The compound library contained 120 natural products obtained from the National Cancer Institute (NCI, Bethesda, MD, USA), and 297 natural products from PI & PI Technology (Guangzhou, Guangdong, China) or The National Center for Drug Screening (Shanghai, China).

### Plasmids

The human APP695 (hAPP) gene was purchased from GeneChem co. Ltd. (Shanghai, China) and cloned into a pcDNA3 vector as described previously^17^. The Nicastrin gene was purchased from Sangon Biotech (Shanghai, China). The cDNA encoding full length human Nicastrin was cloned into the pFastBac1 bacmids with a C-terminal 6×His-FLAG tag. The pcDNA4/TO plasmid (Invitrogen) containing C99-Gal4-VP16 was constructed according to procedures described previously^17^,^30^. The human Notch1-NEXT (Notch 1 extracellular truncation, amino acid residues 1721–2555 in the human sequence) gene was synthesized by GenScript Ltd. (Nanjing, China). The pGL4.31[luc2P/Gal4UAS/Hygro] plasmid was purchased from Promega.

### Cell culture and transfections

HEK293, T-REx HeLa cells and the fibroblast cell line (AG06848) were cultured as described in the Supplementary information. S-20 cells overexpressing human PS1, Flag-Pen-2, Aph-1a2-HA, and NCT-V5/His were cultured as previously described^20^. HEK293 cells were transfected by using X-tremeGENE HP DNA Transfection Reagent according to the manufacturer’s protocol (Roche).

### Stable cell line overexpressing C99-Gal4-VP16 and luciferase

T-REx-HeLa cells (T_100_) stably overexpressing C99-Gal4-VP16 and luciferase were generated according to the method described in refs 17,30. For details, see Supplementary Information.

### Purification of γ-secretase, NCT and C100-Flag

γ-Secretase was purified from S-20 cells as described previously^20^. The recombinant APP-based protein substrate of γ-secretase, namely human C100-Flag, was overexpressed in *E. coli* and purified by using an anti-Flag M2 resin^31^. Full length human NCT was purified as described previously (for details, see Supplementary information)^32^.

### γ-Secretase activity assays

Cell-free *in vitro* γ-secretase assays using the recombinant C100-Flag substrate and purified γ-secretase were performed as described in the Supplementary information^21^.

Cell-based γ-secretase assays were performed using the T_100_ cell line according to the methods detailed in ref. 30 (for details, see Supplementary information).

### Western Blotting and antibodies

Western Blot analysis of full-length APP, APP-CTFs, Notch1-NICD and γ-secretase components was carried out according to the procedures described in the Supplementary information^31^.

### Bicine/urea SDS-PAGE to analyze Aβ38, Aβ40 and Aβ42 from *in vitro* C100-Flag γ-secretase assays

Western blot analysis of the various species of Aβ was performed as described previously^33^, by using the 6E10 antibody.

### Aβ ELISA

Aβ1-x peptides secreted in the cell media were quantitatively measured by ELISA (IBL, Gunma, Japan) according to the standard protocol from the manufacturer. Aβ40 and Aβ42 generated in the C100-Flag γ-secretase assays stopped with 0.5% SDS (final concentration) were quantified with the human Beta Amyloid [1–40] or [1–42] colorimetric ELISA kit, respectively.

### Surface plasmon resonance analysis

Surface plasmon resonance (SPR) with a Biacore T100 (GE Healthcare) was used to investigate the binding of DHEC to γ-secretase or NCT, the largest subunit of γ-secretase. A Biacore sensor Chip NTA that is designed to bind His-tagged proteins was used to immobilize γ-secretase. The SPR assay was performed in a running buffer (10 mM HEPES, 150 mM NaCl in the presence of 1% DMSO, pH 7.4). The purified His-tagged γ-secretase overexpressing human PS1, Flag-Pen-2, Aph-1a2-HA, and NCT-V5/His (see above) was diluted 6 times in DMSO-free running buffer. For each binding curve, the running buffer containing 500 μM NiCl_2_ was first injected to saturate the NTA chip. Then, His-tagged γ-secretase was injected and immobilized on the Ni^2+^ -coated sensor chip. Compounds at the indicated concentrations were injected onto the surface of the sensor chip, and the corresponding binding spectrum was recorded. The sensor chip was regenerated with regeneration buffer (10 mM HEPES, 150 mM NaCl, 350 mM EDTA, pH 8.3). Purified human NCT (100 μg) was immobilized onto the flow cell of a CM5 sensor chip in 10 mM sodium acetate (pH 4.0) by using an amine coupling kit. The SPR assay was performed in HBS-P running buffer (10 mM HEPES, 150 mM NaCl and 0.005% surfactant P20 in the presence of 1% DMSO, pH 7.4). To measure the binding affinity of DHEC for γ-secretase and NCT, the compounds were diluted to the following concentrations in running buffer (for binding to γ-secretase: 0.78, 1.56, 3.125, 6.25, 12.5 and 25 μM; NCT: 1.0, 2.0, 3.125, 5.0, 6.25, 8.0, 10.0 and 12.5 μM). The K_d_ values were determined with Biacore evaluation 3.1 software.

### Statistical analysis and Western Blot quantification

All experiments were performed at least twice in duplicate or triplicate with comparable results, and the data are presented as the means ± sd. Statistical analysis was performed using a one-way or two-way ANOVA with Bonferroni’s multiple comparisons tests, and statistical significance is shown as *(P < 0.05), **(P < 0.01) or ***(P < 0.001). The density of the APP-CTFs, NICD, AICD-Flag and Aβ total bands in the Western blots was quantified with Odyssey software (LI-COR Bioscience, Lincoln, Nebraska, USA).

## Additional Information

**How to cite this article**: Lei, X. *et al.* The FDA-approved natural product dihydroergocristine reduces the production of the Alzheimer's disease amyloid-β peptides. *Sci. Rep.* **5**, 16541; doi: 10.1038/srep16541 (2015).

## Supplementary Material

Supplementary Information

## Figures and Tables

**Table 1:**
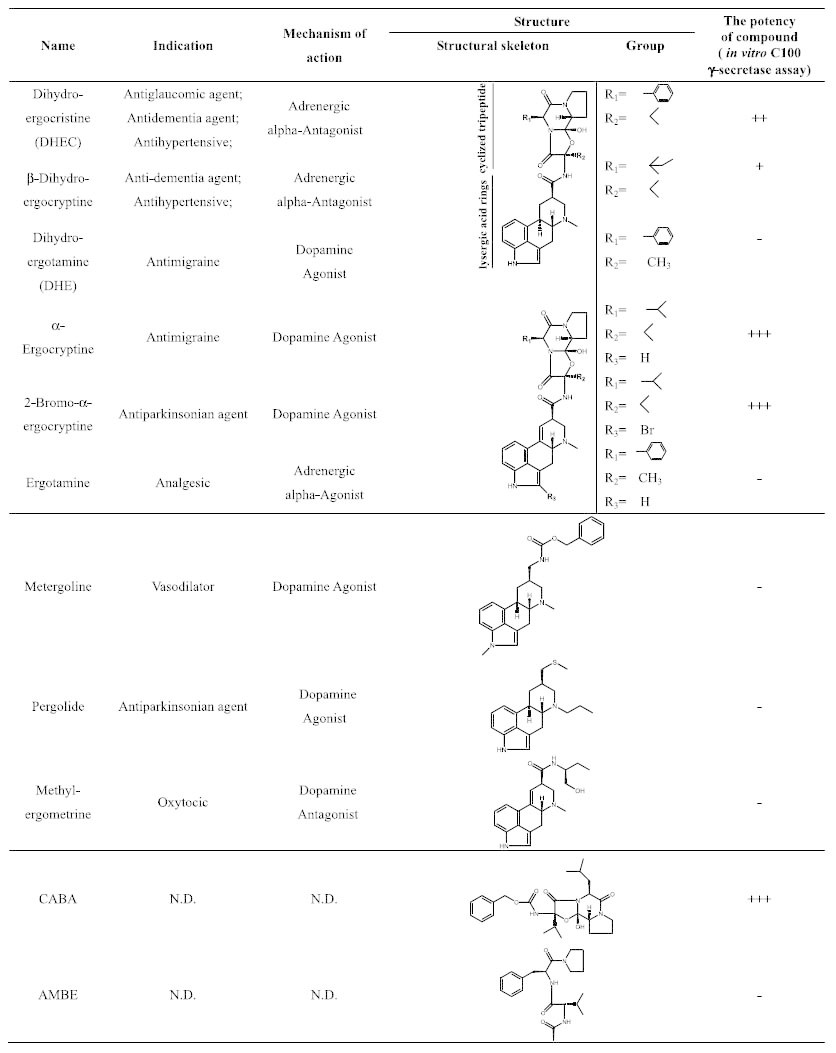
Structural features and treatment indications of compounds used in this study.

The potencies of the tested compounds are indicated (^+ + +^strong inhibitory effect compared to DHEC at the same concentration of 200 µ M; ^+ +^similar inhibitory effect to DHEC at 200 µ M; ^+^weak inhibitory effect compared to DHEC at 200 µ M; ^-^No effect), as estimated by densitometry from the in vitro C100-Flag γ-secretase assay as shown in Fig. 3. N.D. means not determined.
