# Supplementary material for: The FDA-approved natural product dihydroergocristine reduces the production of the Alzheimer’s disease amyloid-β peptides
Source: Sci Rep. 2015 Nov 16;5:16541. doi: 10.1038/srep16541 (PMC4644980; doi:10.1038/srep16541)
Supplement: Supplementary Information [file srep16541-s1.pdf]

Supplementary information

The FDA-approved natural product dihydroergocristine reduces the production of the Alzheimer's disease amyloid-β peptides

Xiling Lei,[1] Jing Yu,[2] Qi Niu,[1] Jianhua Liu,[2] Patrick C. Fraering,[3] and Fang Wu [1,*]

[1]Key Laboratory of Systems Biomedicine (Ministry of Education), Shanghai Center for Systems Biomedicine, Shanghai Jiao Tong University, Shanghai, 200240, China

[2]State Key Laboratory of Microbial Metabolism & School of Life Sciences and Biotechnology, Shanghai Jiao Tong University, Shanghai, 200240, China

[3]Brain Mind Institute, School of Life Sciences, Ecole Polytechnique Federale de Lausanne (EPFL), Lausanne, Switzerland

Methods

Cell culture

HEK293 were maintained in DMEM (Life Technologies), 10% fetal bovine serum (FBS; Life Technologies), and 1% (w/v) penicillin and streptomycin (Life Technologies) in a humidified 5% CO_2 atmosphere at 37°C as previously described.[1] T-REx Hela cells were purchased from Invitrogen and cultured in DMEM supplemented with 10% FBS, 1% P/S and 5 μg/ml blasticidin. The fibroblast cell line (AG06848) from an Alzheimer's disease patient carrying the PS1 missense mutation A246E, was obtained from the Coriell Institute for Medical Research (Camden, NJ, USA) and cultured according to the repository protocols.

Stable cell line overexpressing C99-Gal4-VP16 and luciferase

T-REx-Hela cells were grown in a 6-well plate until 90% confluency was reached before transfection with pcDNA4/TO C99-Gal4-VP16 (1.7 μg) and pGL4.31 [luc2P/Gal4UAS/Hygro] (1.7 μg) plasmids by using the Lipofectamine 2000 (Invitrogen) transfection reagent. Single colonies of transfected cells were obtained using the method of limiting dilution and then cultured in DMEM supplemented with 10% FBS, 1% P/S, 250 μg/ml hygromycin, 200 μg/ml zeocin, and 5 μg/ml blasticidin (final concentration). The stable clones were screened and selected by using tetracycline and DAPT, a known inhibitor of γ-secretase, as described previously,[2]

and resulted in a highly sensitive stable cell line (T_{100}) for quantitatively monitoring the activity of γ-secretase.

Purification of NCT

Recombinant bacmids were used to transfect Sf9 cells to make the baculovirus, which was then used to infect High Five suspension insect cells (Invitrogen). After three cycles of freezing and thawing, the cell lysates were centrifuged and first purified by the Ni^{2+}-NTA affinity chromatography (Qiagen), with the binding buffer containing 25 mM Tris (pH 8.0), 300 mM sodium chloride and 20 mM imidazole, and eluted with buffer containing 25 mM Tris (pH 8.0), 300 mM sodium chloride and 500 mM imidazole. The eluted fractions were further purified by Source 15Q anion exchange chromatography (GE healthcare), equilibrated with buffer A containing 25 mM Tris (pH 8.0) and 2 mM dithiothreitol, and eluted with buffer A plus a linear sodium chloride concentration gradient of 0–500 mM. The proteins were further purified by the Superdex200 gel filtration chromatography (GE Healthcare). The Superdex200 buffer contained 10 mM Tris–HCl (pH 8.0), 250 mM sodium chloride, 2 mM dithiothreitol and 1 mM EDTA. Peak fractions were combined.

γ-Secretase activity assays

γ-Secretase purified from S-20 cells was solubilized in 0.2% (w/v) CHAPSO in 50 mM HEPES buffer (pH 7.0) containing of 150 mM NaCl, 5 mM $MgCl_2$ and 5 mM $CaCl_2$, and incubated with 0.1% (w/v) PC and 1 μM or otherwise indicated

concentrations of substrate at 37 °C for 4 h, after which the enzymatic reactions were stopped by adding 0.5% SDS (final concentration). The samples were analyzed by Western Blot for AICD-Flag or Aβ.

T_{100} cells were seeded in 96-well plates (5×10^5 cells/well) for a day, and incubated with 1 μg/ml tetracycline in the presence or absence of the tested compounds. After 24 h of incubation, cells and media were collected for luciferase activity and cell viability assays, respectively. For luciferase activity assays, cells were lysed for 5 min in 100 μl Glo lysis buffer and the luciferase activity was measured by mixing 90 μl of each lysate and Bright-Glo luciferase assay reagent in 96-well Nunc MaxiSorp plates (Nunc, Roskilde, Denmark). Following 5 min of incubation at room temperature, the luminescence emitted in individual wells was monitored by using a microplate reader (Synergy2, BioTek, Winooski, VT, USA). The primary screen was performed by testing the natural product library at 10 μM, and compounds showing more than 40% inhibition were selected for dose-dependent inhibition studies for γ-secretase activity in T_{100} cells. DAPT (1 μM) was included in each plate as a positive control.

Cytotoxicity assay

The effect of each compound on cell viability was examined by using the CytoTox-One^{Tm} kit (Promega) according to the manufacturer's instructions. Briefly, Cells were seeded in a 96-well plate for 1 day before treatment with the indicated compounds overnight. The media (50 μl) from the treated cells were transferred into 96-well plates, mixed with 50 μl of CytoTox-One^{Tm} substrate solution and incubated

for 10 min before adding 25 μl of the stop solution. The fluorescence was monitored with a microplate reader (emission at 590 nm after excitation at 560 nm).

Western blot and antibodies

WT HEK293 or fibroblast cells were collected and lysed in 1% Nonidet P-40-HEPES buffer, and equal amounts of proteins [normalized by BCA (Pierce)] were separated by SDS-PAGE onto a 12% Tris-glycine gel, transferred onto a PVDF membrane (Bio-Rad, USA) and further probed with antibody CT15 (for APP/APP-CTFs, 1:2000, Sigma-Aldrich), MAB1563 (1:1000; Millipore, Milford, MA, USA) or ab134195 (1:1000; Abcam, Cambridge, MA, USA) for PS1-NTF, UD-1 (for Pen-2, 1:500; a gift of Dr. Helena Karlström, Karolinska Institute, Sweden), NCT164 (for NCT, 1:1000; BD Bioscience, Bedford, MA, USA) as well as with an antibody for β-actin (1:2000, Abmart). For the analysis of Notch NICD, cell extracts were separated by SDS-PAGE onto a 10% Tris-glycine gel, transferred onto a PVDF membrane and blotted for NICD with an anti-Notch Ab1744 antibody (for the free N-terminus of NICD, 1:500, Cell Signaling Technology, Beverly, MA, USA).

Samples from *in vitro* γ-secretase activity assays were run on 16% Tricine-SDS-PAGE gels and transferred onto PVDF to detect Aβ and AICD-Flag with 6E10 (1:1000, Covance, Berkeley, CA, USA) and CT15 antibodies (1:5000, Sigma-Aldrich), respectively.[3]

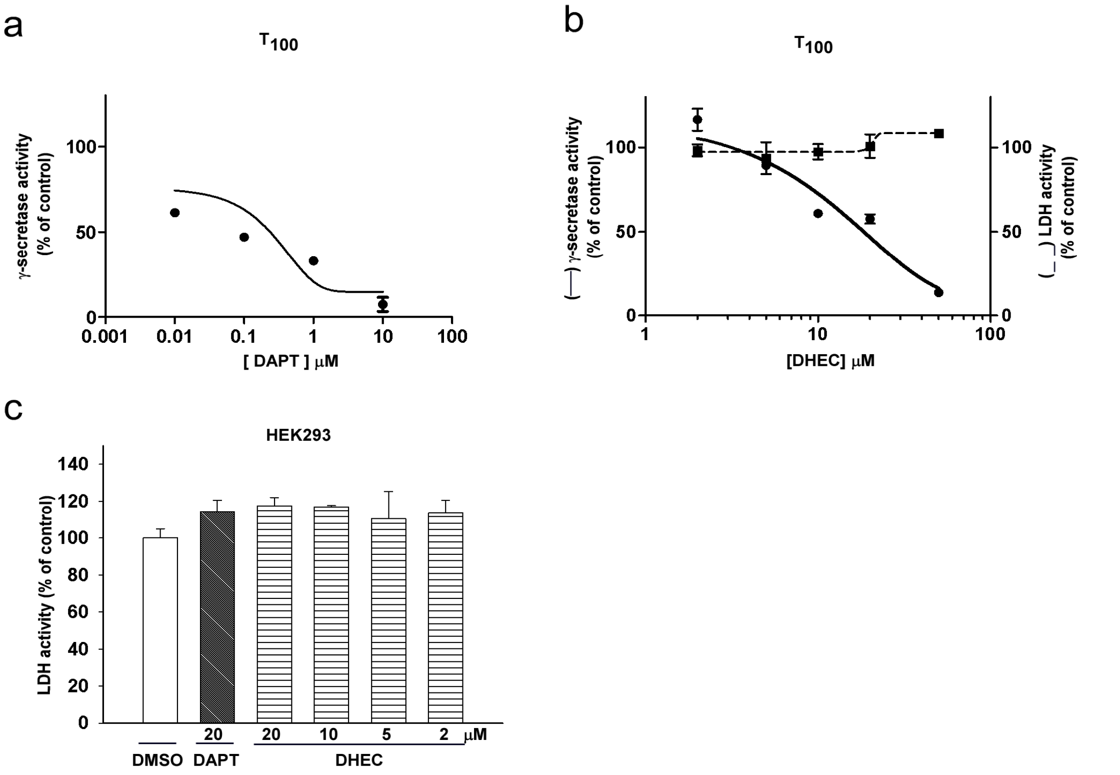

Figure S1 Effects of dihydroergocristine and DAPT in T_{100} cells and HEK293 cells. (**a**) Effects of DAPT on the activity of γ-secretase in T_{100} cells. T_{100} cells were incubated with DMSO (control, 100%) or DAPT at the indicated concentrations for 24 h, collected and then lysed before measuring γ-secretase activity (Supplementary methods). Data are presented as mean ± SD. (n=3). (**b**) Effects of dihydroergocristine (DHEC) on γ-secretase and LDH activity in T_{100} cells. T_{100} cells were incubated with DMSO (control, 100%) or DHEC at the indicated concentrations for 24 h. After lysis of the treated cells, both lysates and medium were collected to measure the activity of γ-secretase (—) or LDH (--) as described in Supplementary methods. Data are presented as mean ± SD. (n=3). (**c**) Effects of DHEC on LDH activity in HEK293 cells. Cell media from DAPT or DHEC -treated HEK293 cells (Figure 1a), were collected to measure LDH activity (Supplementary methods). Data are presented as mean ± SD.

(n=3).

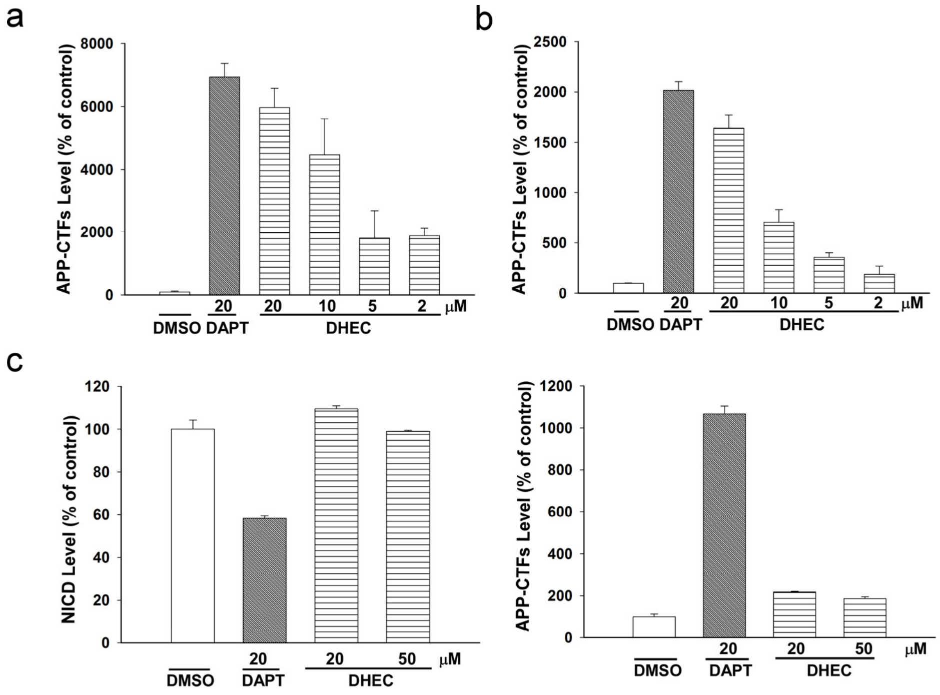

Figure S2 Densitometric quantification with the Odyssey software of the APP-CTFs and NICD bands shown in the Figure 1. Data are expressed as percentage of control (DMSO, 100%) and presented as mean ± SD. (n=2).

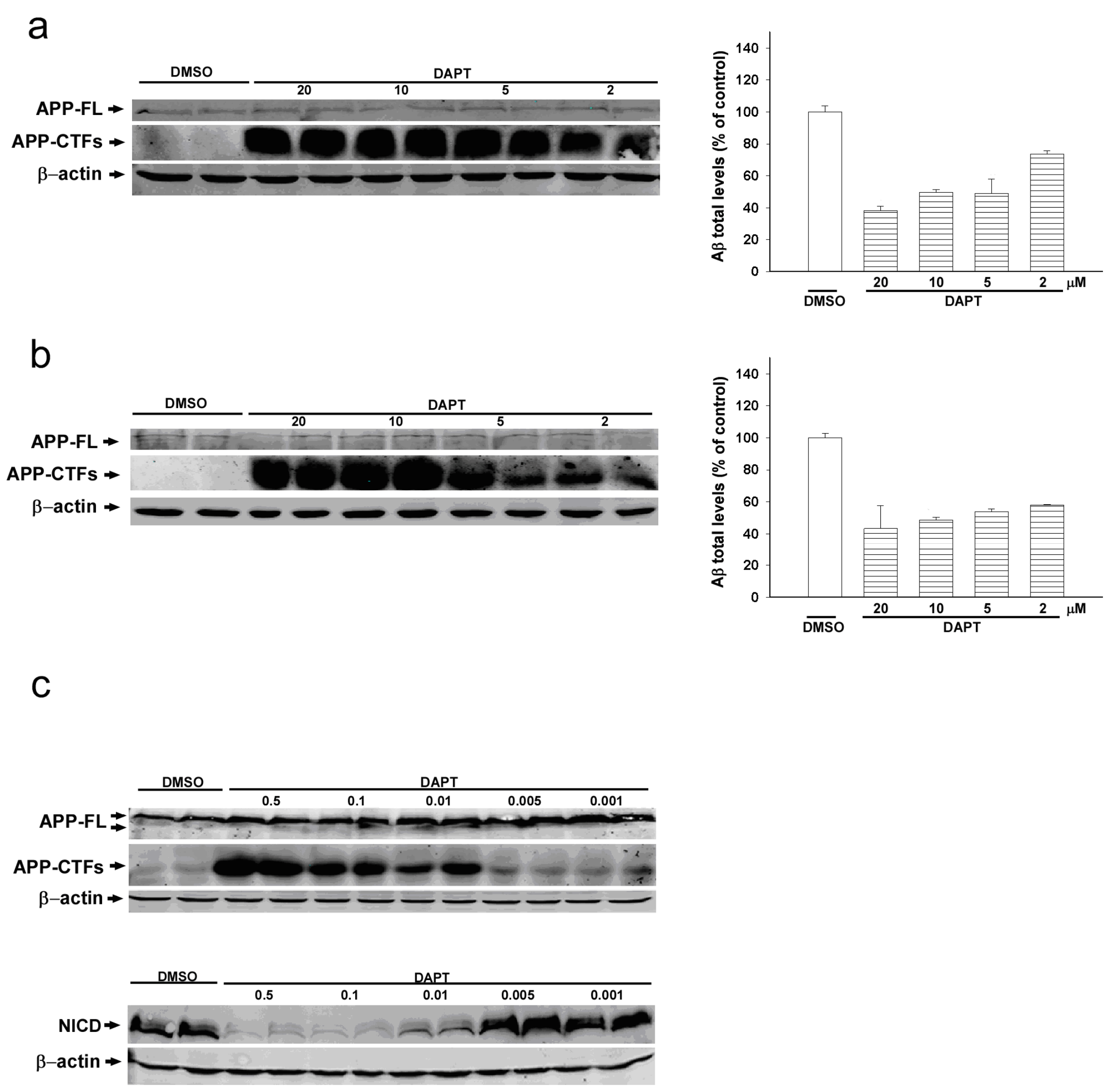

Figure S3 Effects of DAPT on γ-secretase activity in cells. (**a**) Effects of DAPT on endogenous APP-CTFs and secreted Aβ from HEK293 cells. HEK293 cells were incubated with DMSO (control) or DAPT at the indicated concentrations (μM) in 24-well plates for 24 h. Cells were collected and lysed in Nonidet P-40-HEPES buffer, and equal amounts of protein (normalized by BCA) were loaded onto a 12% Tris-glycine SDS-PAGE and blotted for APP-FL and APP-CTFs (CT15, left panel). Levels of β-actin were used as a loading control. The corresponding media were collected, and total Aβ was measured by ELISA (right panel, means ± SD.; n≥2). (**b**)

Effects of DAPT on endogenous γ-secretase activity in fibroblast cells from an AD patient. The fibroblast cells were treated with various compounds at the indicated concentrations (μM), and the corresponding APP-FL, APP-CTFs and β-actin levels, as well as total Aβ were measured as described above. Means ± SD ($n \geq 2$). (**c**) Effects of DAPT on the cleavage of human APP or Notch1-NEXT in HEK293 cells overexpressing hAPP or Notch1-NEXT. HEK293 cells were first transiently transfected for 24 hours with plasmids encoding for hAPP or Notch1-NEXT, and further incubated with DAPT at the indicated concentrations (μM) for an additional day, before being lysed in 1% Nonidet P-40-HEPES buffer. Cell supernatants were separated by SDS-PAGE onto 12% and 10% Tris-glycine gels and blotted for APP-CTFs (CT15) and NICD (Ab1744), respectively. Levels of β-actin served as equal loading controls.

a

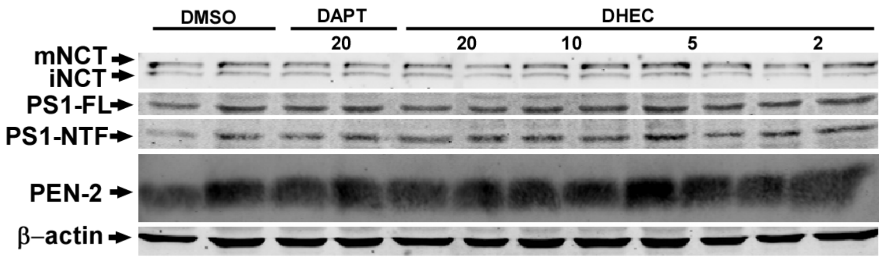

b

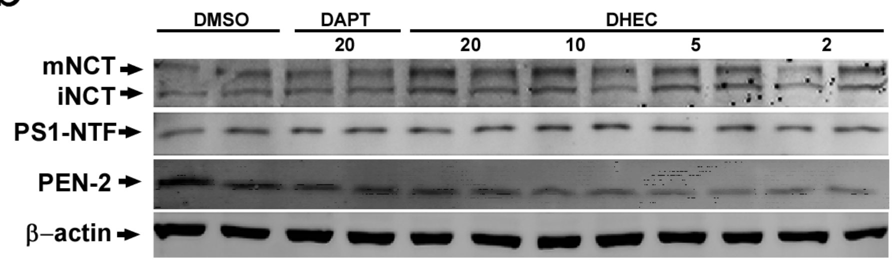

Figure S4 Intracellular effects of DHEC on endogenous γ-secretase subunits. (**a**) Effects of DHEC on endogenous γ-secretase subunits in HEK293 cells. Protein extracts from HEK293 cells treated with DMSO, DAPT or DHEC (μM) were separated by SDS-PAGE onto a 12% Tris-glycine gel and blotted for γ-secretase subunits: Nicastrin (NCT164), full length PS1 or PS1-NTF (MAB1563), and Pen-2 (UD-1). Levels of β-actin served as equal loading controls. (**b**) Effect of DHEC on subunits of endogenous γ-secretase in fibroblast cells. Whole cell extracts from fibroblast cells treated with DMSO, DAPT or DHEC (μM) were separated on a 12% Tris-glycine SDS-PAGE and blotted for γ-secretase subunits: nicastrin (NCT164), PS1-NTF (ab134195), and Pen-2 (UD-1). Levels of β-actin served as equal loading controls.

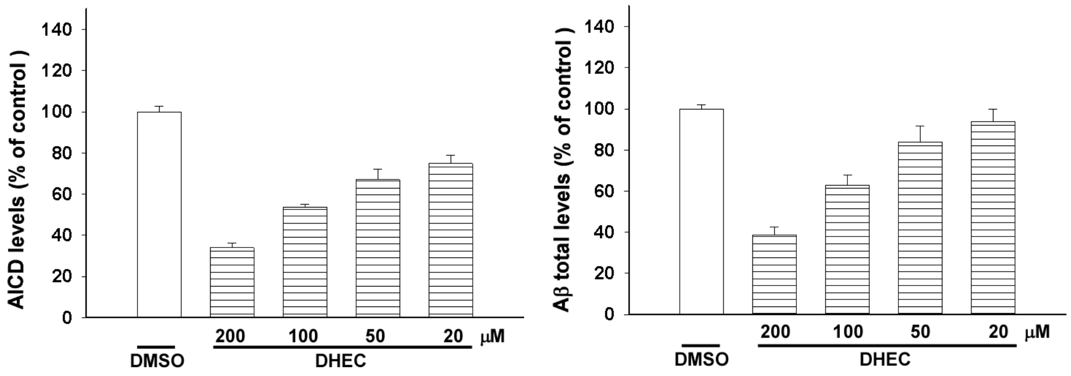

Figure S5 Effects of DHEC on the processing of human APP C100-Flag.

The AICD-Flag and total Aβ bands shown in Figure 2a were quantified by densitometry using the Odyssey software.

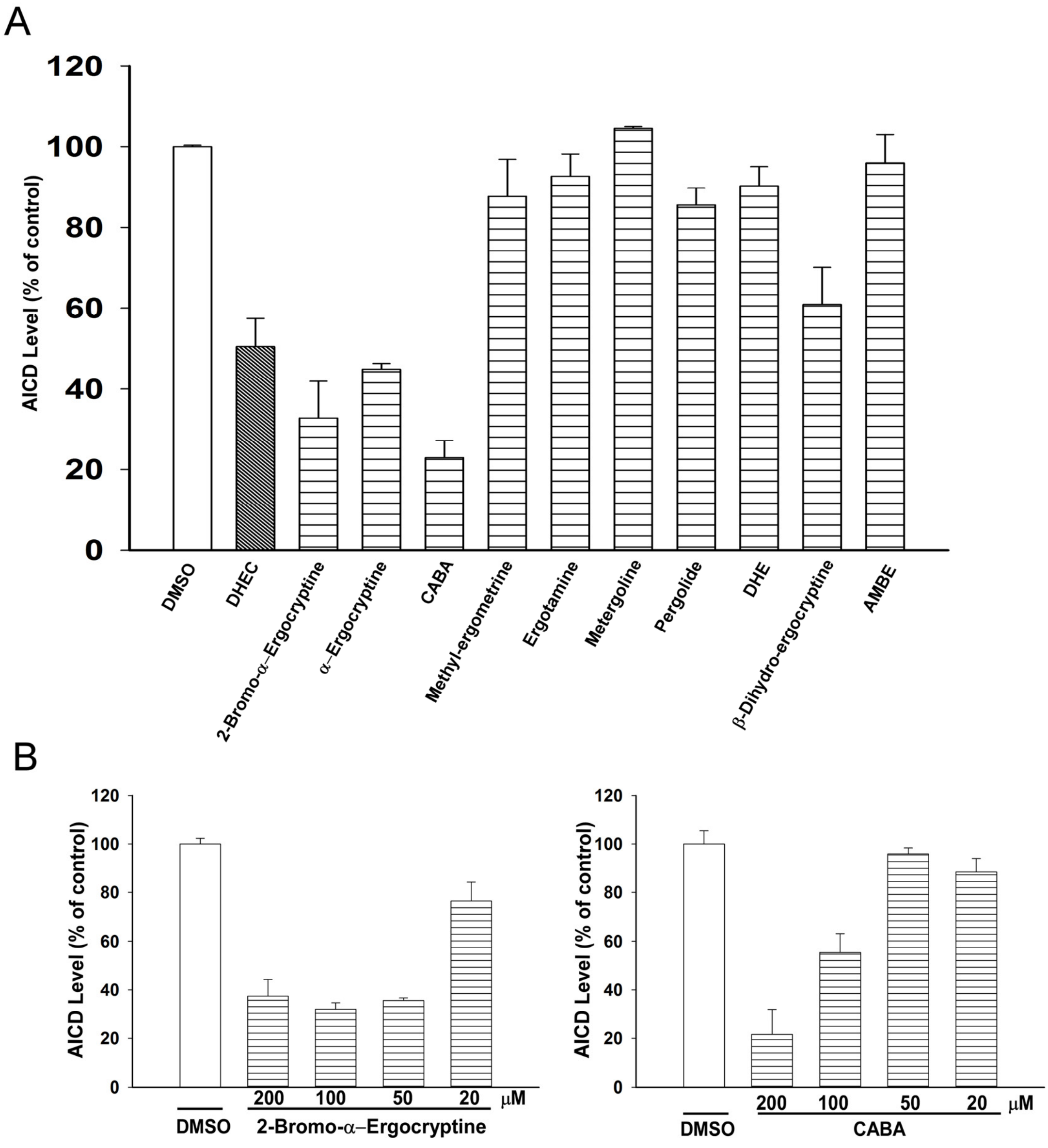

Figure S6 The AICD bands shown in the Figures 3a and 3b were quantified by densitometry using the Odyssey software. Data are expressed as percentage of control (DMSO, 100%) and presented as mean ± SD. (n=2).

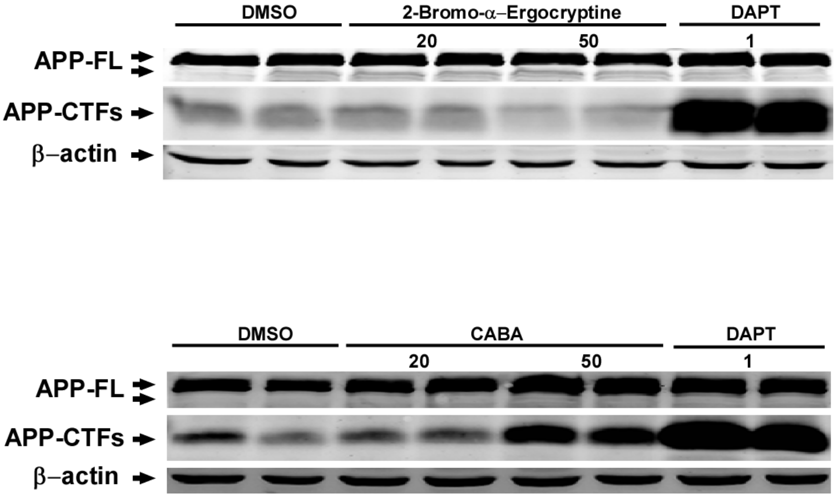

Figure S7 Effects of 2-bromo-α-ergocryptine and CABA on the cleavage of hAPP in cells. HEK293 cells were first transiently transfected with hAPP for 24 hours, further incubated with 2-bromo-α-ergocryptine or CABA at the indicated concentrations (μM) for an additional day, and finaly lysed in 1% Nonidet P-40-HEPES buffer. Cell lysates were separated by SDS-PAGE onto a 12% Tris-glycine gel and Western blotted for APP-CTFs (CT15). Levels of β-actin served as equal loading controls.

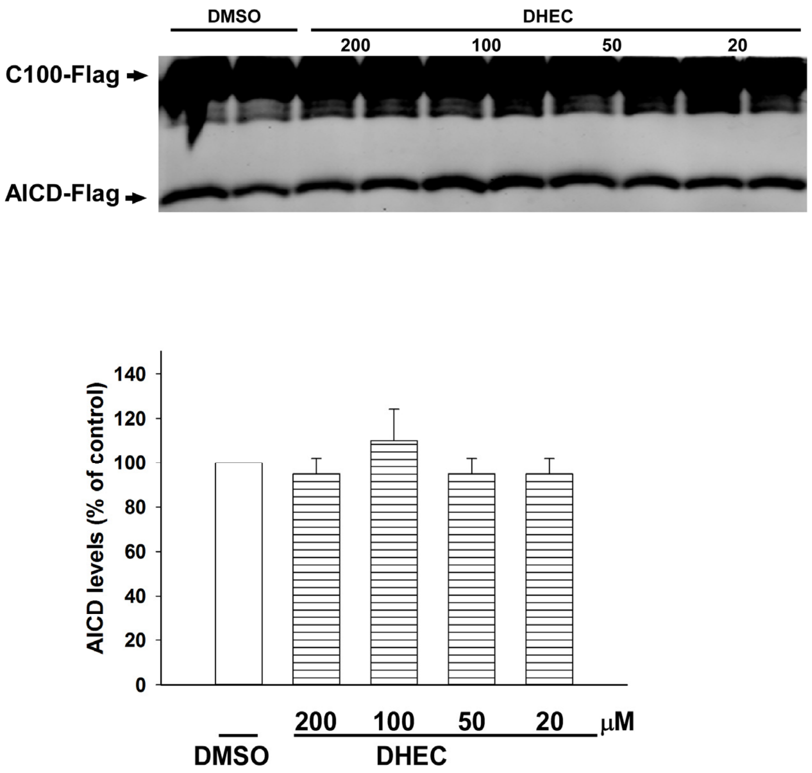

Figure S8 Increased concentration of APP-C100 substrate attenuates the inhibitory effect of DHEC in cell-free γ-secretase assays. Purified γ-secretase was incubated at 37°C for 4 h with 4 μM C100-Flag substrate, 0.1% PC, and with the indicated concentrations of dihydroergocristine (DHEC, μM) or DMSO (control, 100%). Reactions were stopped by adding 0.5% SDS (final concentration) and the resulting products were separated by SDS-PAGE onto a 16% Tricine gel and detected with anti-AICD-Flag antibody (CT15). The AICD-Flag bands showed in the upper panel were quantified by densitometry using the Odyssey software (lower panel).

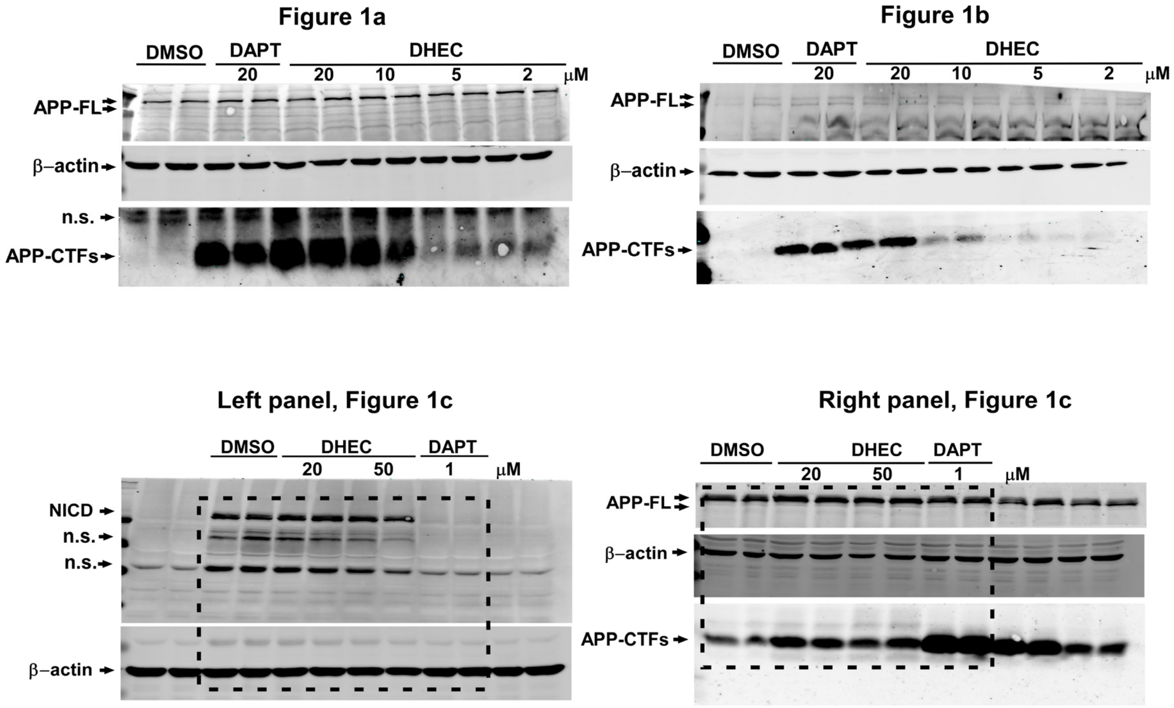

Figure S9 Full blots of Figure 1. The dash box containing region in the blot is corresponding to the present data. n.s. indicates the non-specific bands.

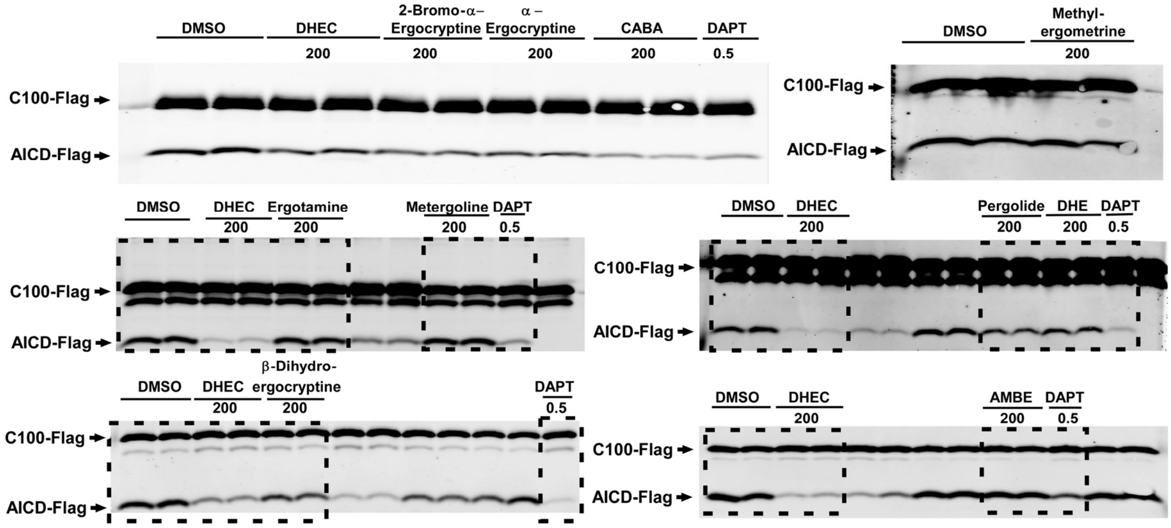

Figure S10 Full blots of Figure 3a. Data reported in the Figure 3a are highlighted in each blot by dashed boxes.

References:

1. Haass, C., Capell, A., Citron, M., Teplow, D.B. & Selkoe, D.J. The vacuolar H(+)-ATPase inhibitor bafilomycin A1 differentially affects proteolytic processing of mutant and wild-type beta-amyloid precursor protein. *J. Biol. Chem.* **270**, 6186-6192 (1995).
2. Liao, Y.F., Wang, B.J., Cheng, H.T., Kuo, L.H. & Wolfe, M.S. Tumor necrosis factor-alpha, interleukin-1beta, and interferon-gamma stimulate gamma-secretase-mediated cleavage of amyloid precursor protein through a JNK-dependent MAPK pathway. *J. Biol. Chem.* **279**, 49523-49532 (2004).
3. Fraering, P.C. et al. gamma-Secretase substrate selectivity can be modulated directly via interaction with a nucleotide-binding site. *J. Biol. Chem.* **280**, 41987-41996 (2005).
